# Antitumor effect of resveratrol on chondrosarcoma cells via phosphoinositide 3-kinase/AKT and p38 mitogen-activated protein kinase pathways

**DOI:** 10.3892/mmr.2021.12238

**Published:** 2021-06-23

**Authors:** Zixun Dai, Pengfei Lei, Jie Xie, Yihe Hu

Mol Med Rep 12: 3151-3155, 2015; DOI: 10.3892/mmr.2015.3683

Following the publication of this paper, it was drawn to the Editors' attention by a concerned reader that the cell apoptotic data shown in Fig. 2 were strikingly similar to data appearing in different form in other articles by different authors. Owing to the fact that the contentious data in the above article had already been published elsewhere, or were already under consideration for publication, prior to its submission to *Molecular Medicine Reports*, the Editor has decided that this paper should be retracted from the Journal. After having been in contact with the authors, they agreed with the decision to retract the paper. The Editor apologizes to the readership for any inconvenience caused.

